# Supplementary material for: Menstrual hygiene management practices and associated health outcomes among school-going adolescents in rural Gambia
Source: PLoS One. 2021 Feb 25;16(2):e0247554. doi: 10.1371/journal.pone.0247554 (PMC7906402; doi:10.1371/journal.pone.0247554)
Supplement: S1 File — (DOCX) [file pone.0247554.s001.docx]

**
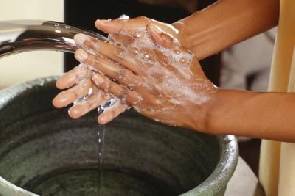
Protocol for collecting urine**

Wash your hands well with soap and water


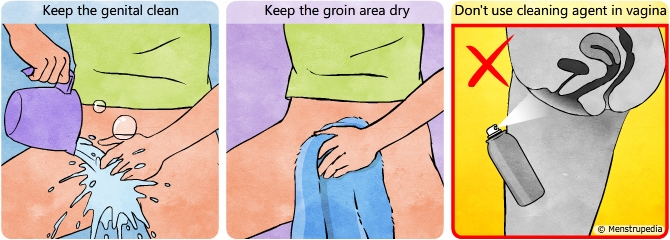


Wash the genital area Dry it well

Clean your genital area well with water and then dry it

Remove the lid of the container, taking care not to touch the inside of the container.


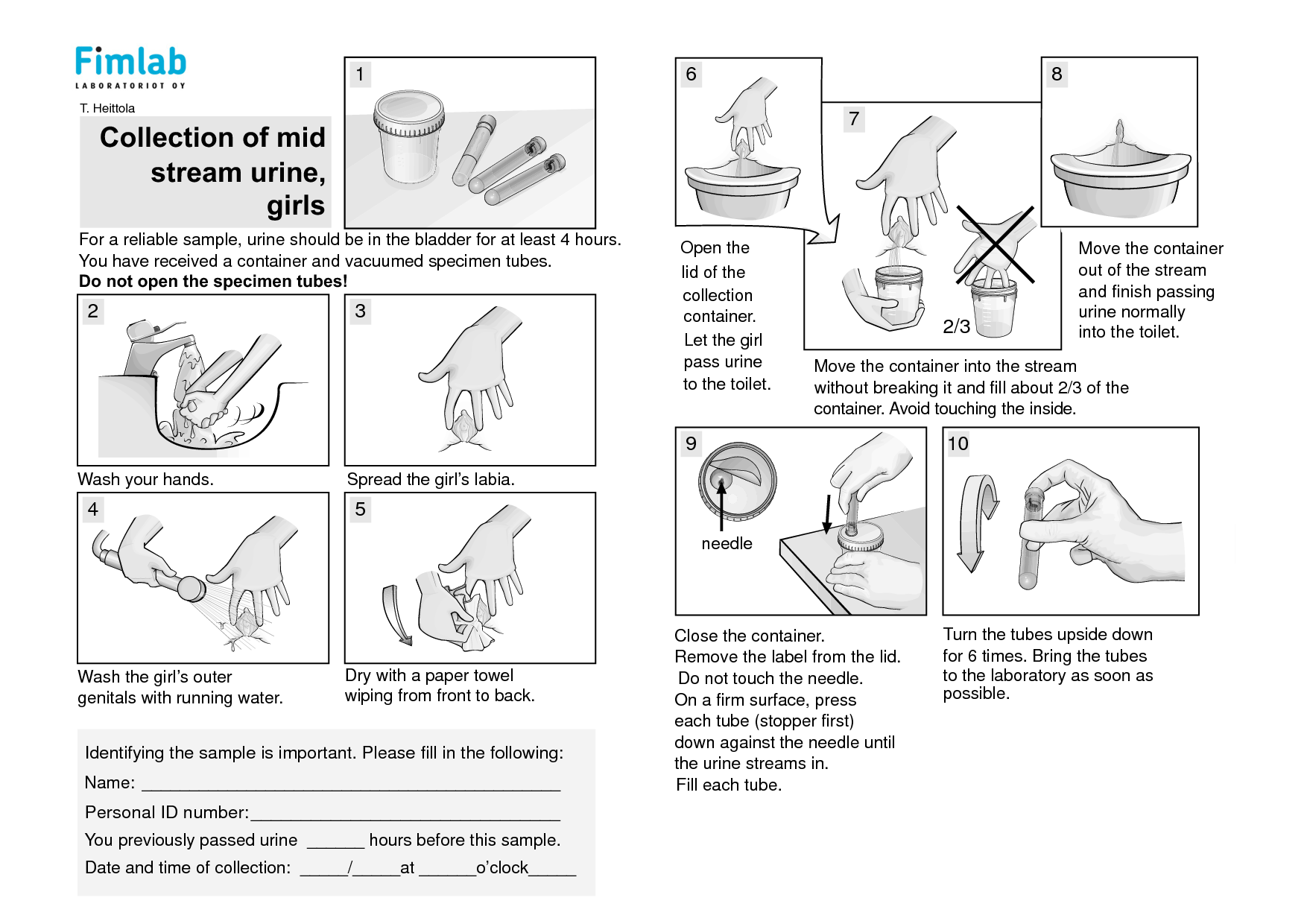


Urinate into the toilet for a few seconds


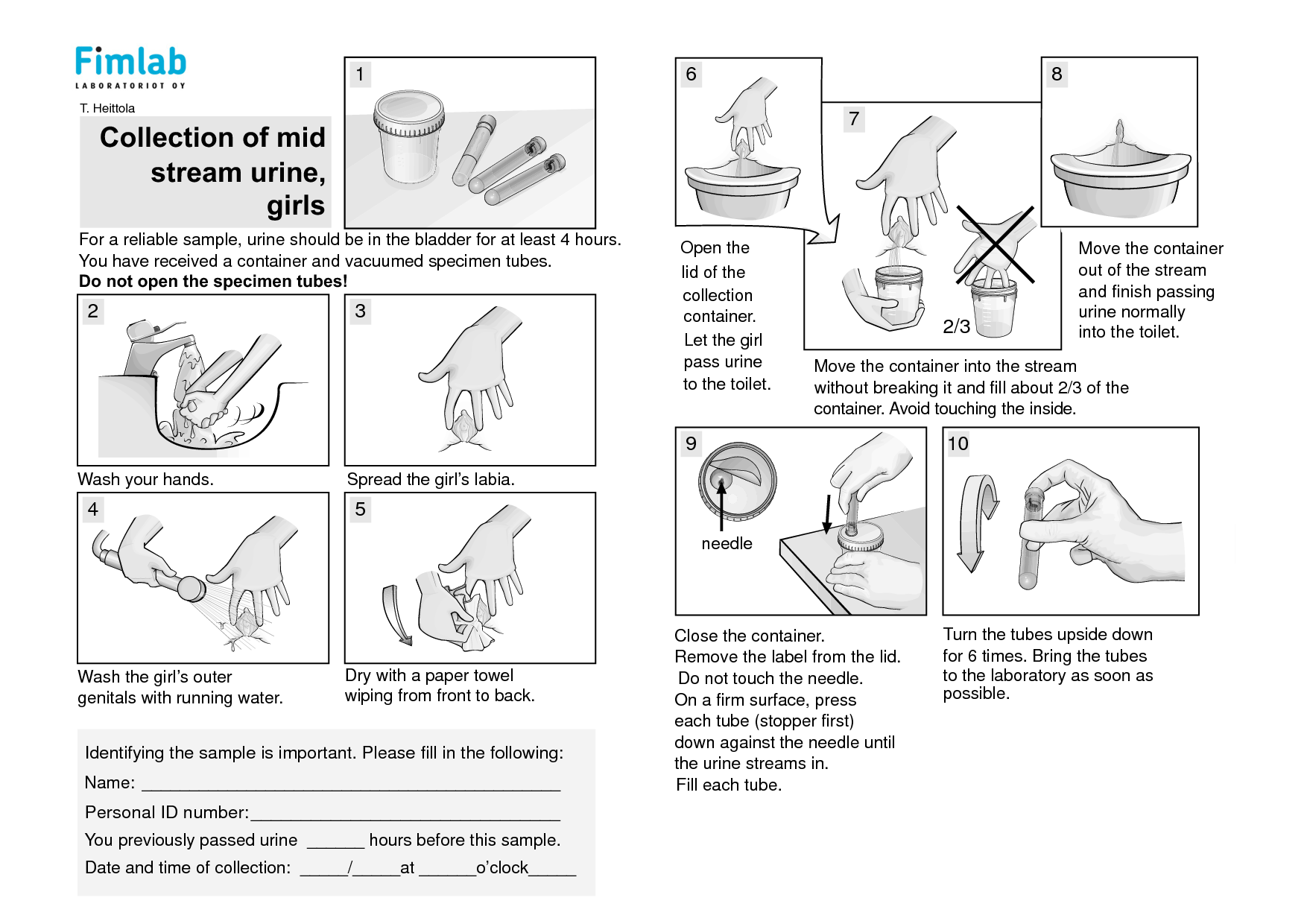


Then Urinate in the container

Finish urinating in the toilet


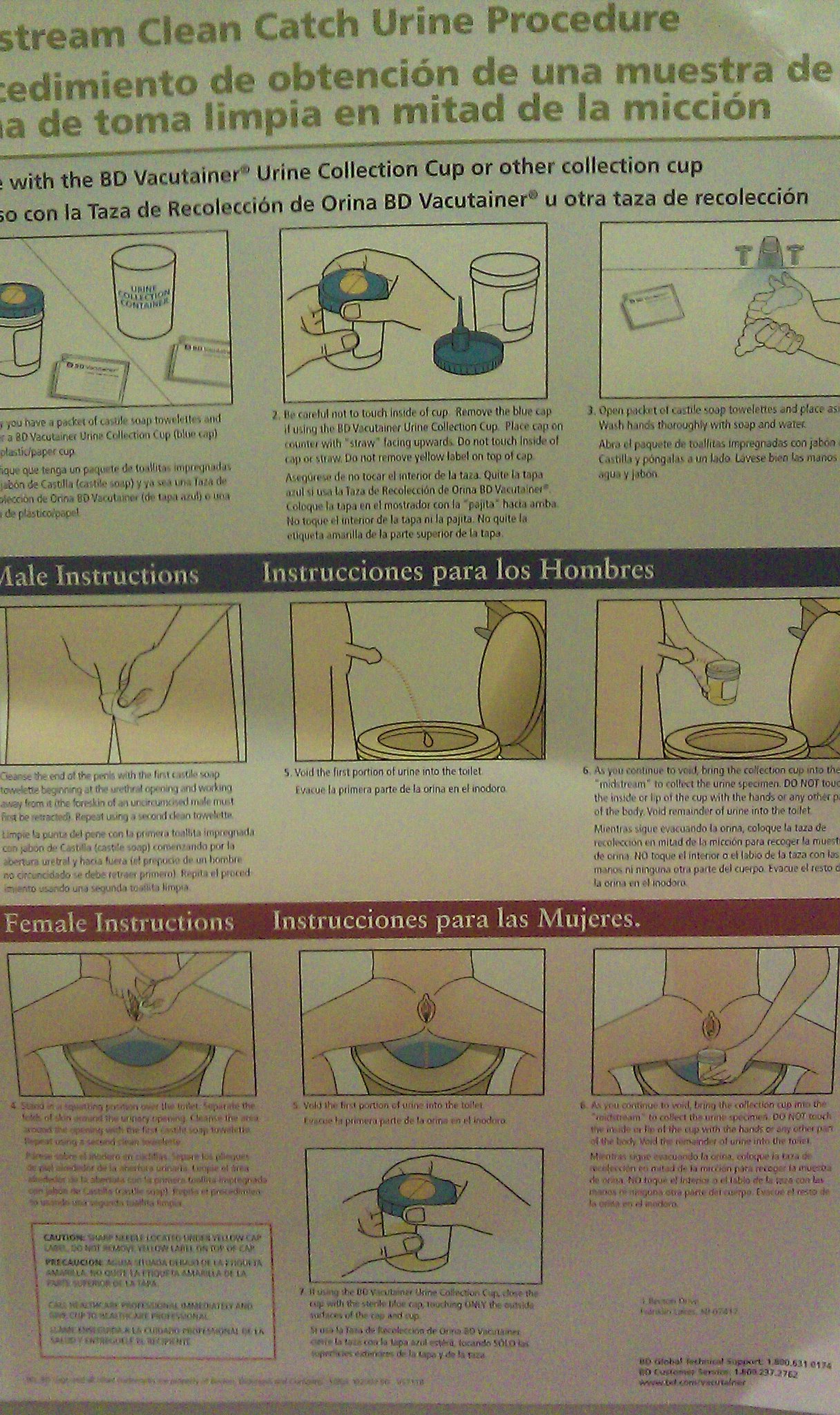


Tightly screw the lid on to the urine container, again taking care not to touch the inside of the container.


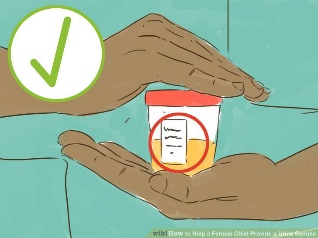


Place the container in the bag provided and hand it back to the research team.


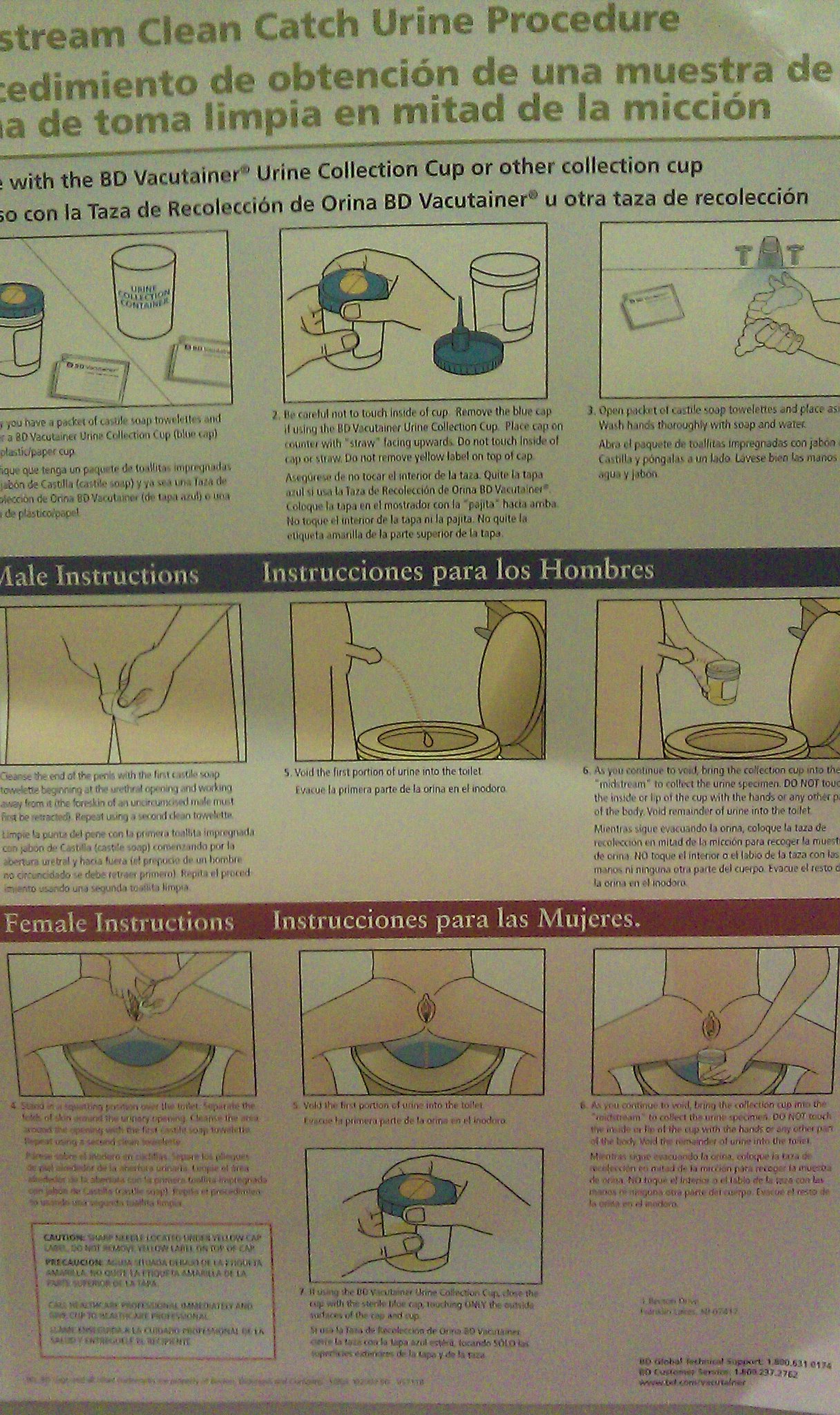


Wipe yourself well Pee a little in the toilet Then continue peeing in the cup

**Urine dipstick field testing protocol**

Combur-Test® Strips protocol:

1. Dip the test strip in urine for about 1 second.


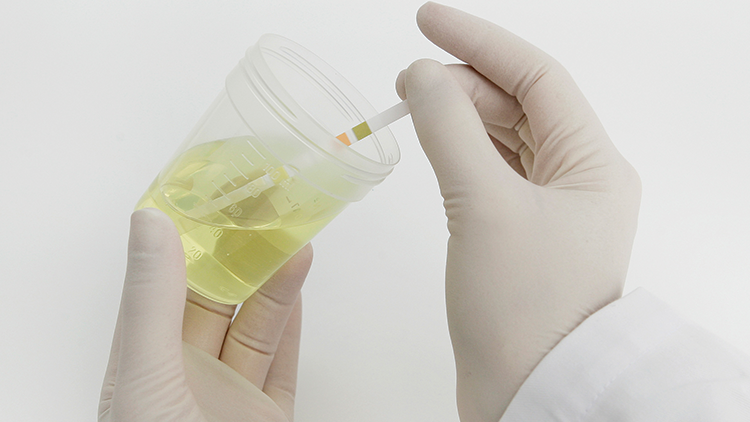


1. Wipe the edge against the rim of the vessel to remove excess urine when withdrawing the strip.


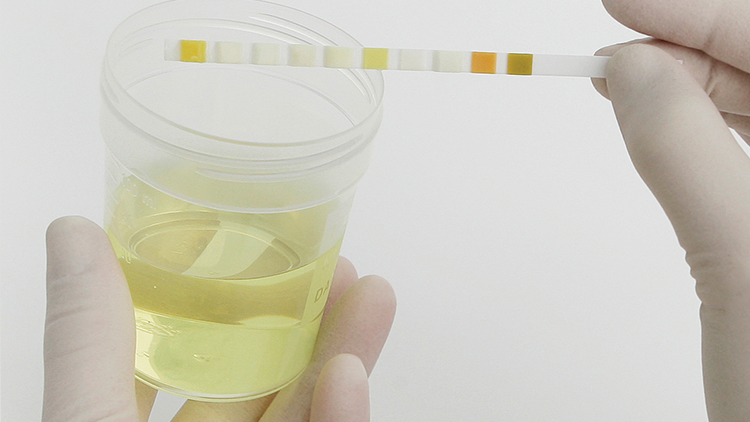


1. After 60 seconds, compare the colour of the detection pad of the strip with the colour scale on the test strip vial. Strips face downwards during operation and fingers stay clear and safe from the samples.


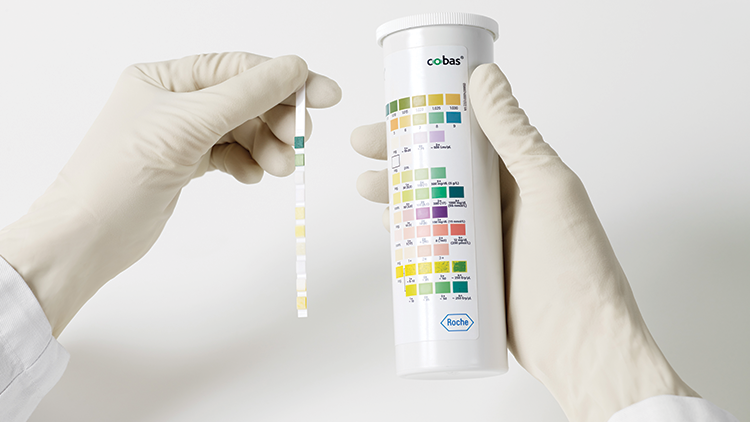


**IMPORTANT:** Ensure samples are tested within an hour and half of collection.
