# Supplementary material for: Menstrual hygiene management practices and associated health outcomes among school-going adolescents in rural Gambia
Source: PLoS One. 2021 Feb 25;16(2):e0247554. doi: 10.1371/journal.pone.0247554 (PMC7906402; doi:10.1371/journal.pone.0247554)
Supplement: Attachment — Submitted filename: Nabwera et al_Response to Reviewers_15Oct2020_Final.docx [file pone.0247554.s003.docx]

15^th^ October 2020

Peter F.W.M. Rosier, M.D. PhD

Academic Editor

PLOS ONE

Dear Dr Rosier,

**RE: Manuscript reference number: PONE-D-20-15732**

**Title: “Menstrual hygiene management practices and associated health outcomes among school-going adolescents in rural Gambia."**

Thank you for your e-mail from the 4^th^ September 2020, requesting us to revise our manuscript to incorporate the editorial and reviewers’ comments. Please find below a point-by-point response to all yours and your reviewers’ comments. We have indicated where changes have been made in the text, using page and line numbers of the “tracked changes” version. Also find uploaded the revised manuscript and supplementary material. We have also uploaded a clean copy. I trust that we have addressed all your comments adequately and that you would kindly consider publishing our manuscript in your journal.

**Editorial comments:**

In your revised cover letter, please address the following prompts:

*a) If there are ethical or legal restrictions on sharing a de-identified data set, please explain them in detail (e.g., data contain potentially identifying or sensitive patient information) and who has imposed them (e.g., an ethics committee). Please also provide contact information for a data access committee, ethics committee, or other institutional body to which data requests may be sent.*

There are no ethical or legal restrictions on sharing a de-identified data set. Data access requests can be sent to Dr Belen Torondel [Belen.Torondel@lshtm.ac.uk](mailto:Belen.Torondel@lshtm.ac.uk)

b) *If there are no restrictions, please upload the minimal anonymized data set necessary to replicate your study findings as either Supporting Information files or to a stable, public repository and provide us with the relevant URLs, DOIs, or accession numbers. Please see* [*http://www.bmj.com/content/340/bmj.c181.long*](http://protect-eu.mimecast.com/s/mrTFCY58PILxnLkT3okh5?domain=bmj.com) *for guidelines on how to de-identify and prepare clinical data for publication. For a list of acceptable repositories, please see* [*http://journals.plos.org/plosone/s/data-availability#loc-recommended-repositories*](http://protect-eu.mimecast.com/s/KG7LCZ01Qi5Nk5PuNUK5B?domain=journals.plos.org)*.*

Data will be available as Supporting Information file.

*We will update your Data Availability statement on your behalf to reflect the information you provide.*

 Thank you.

4. *Your ethics statement must appear in the Methods section of your manuscript. If your ethics statement is written in any section besides the Methods, please move it to the Methods section and delete it from any other section. Please also ensure that your ethics statement is included in your manuscript, as the ethics section of your online submission will not be published alongside your manuscript.*

**Authors:** The ethics statement information is already in the Method section.

**Reviewers’ comments:**

**Reviewer #1: Overall a good topic to address and needed research.**

*1. However conceptually it is hard to follow as there are too many outcomes and no focused message as a result. The results are also simply described and plausibility of associations are not explained. I believe a major revision is needed.

I recommend to refine the study by focusing on the messages in the concluding paragraph and then presenting data that supports (or not) those objectives and explaining them more in depth. Much of the data presented could be removed in order to have a more coherent message.*

**Authors:** We understand the concerns of the reviewer, however we believe it is important to present all the outcome data available in this study, as there is a scarcity of information about this topic in Gambia. Our study was exploratory, seeking to describe adverse health outcomes associated with poor menstrual hygiene management among adolescent girls in a rural context of The Gambia. We therefore focussed on 3 main conditions that the literature suggested were associated with poor menstrual hygiene management in other low-and middle-income countries i.e. urinary tract infections (using common symptoms and urine dipstick); reproductive tract infections (common symptoms) and depression (modified Beck depression inventory). We appreciate your comments, and they have been very useful to help us revise the manuscript and clarify which outcomes we are studying along the different manuscript sections (Abstract, objectives, methods). We have also rewritten some paragraphs of the results and discussion section and provided more clear explanation of plausibility of associations of the different risk factors identified and the different outcomes. Hope the manuscript is clearer now.

*2. Technically, it is unclear if the study instruments for data collection were validated. If so, please describe or reference.*

**Authors**: Thanks for your comment, and to notice that we did not include information about how we validated our data collection tools. We have added a small sub-section explaining how we tested and validated our study instruments (Page 7, lines 7-25; Page 8, lines 1-5).

*3. Model selection process is not explained, nor are the co-variables.*

**Authors:** In page 8, line 7-13 we have described the model selection process in more detail and provided more information on where the details of the co-variables for each of the 3 multivariable models can be found. There was one model for each of the three health outcomes ((i.e. depression, UTI, RTI). For each model explanatory variables that were statistically significant in the univariable analysis as well as those that had biologically plausible associations with each of the three health outcomes were included.

*4. Please report the rate of missing data in the final model.*

**Authors:** Rates of missing data have been included in the footnote of Tables 1 (Sociodemographic) & Table 3 (Menstrual Hygiene Management). We have also included the details of the missing data in each of the models. (Page 9, line 22-24)

**Reviewer #2: Questions for the authors from the reviewer**

*1. Aims and objectives of the study were not stated clearly in the manuscript?*

**Authors**: Thanks for your comment, we have added a clearer aim and objective of the study at the end of the introduction section, hope now the objectives are clear. (Page 4, lines 1-6)

*2. Using an estimated rate of prevalence of urogenital infection of 27%, use of reusable cloth of 40%, prevalence of drying inside the house of 21.6% and prevalence of school absenteeism of 23%. Based on these estimates and using precision/absolute error of 5% and a type 1 error of 5%, a sample size of 368 was calculated. – Which one of the estimates was used in calculating the sample size? Explain the calculation in the methodology?*

**Authors**: Thanks for your comment, we forgot to include that we used 40% prevalence estimated as this resulted in the larger sample size. We have included clarification in the sample size section.

*3.* *Girls who were pregnant were excluded – How was it ensured?*

**Authors**: Thank you for your comment. During participant recruitment the field assistants asked the girls if they were pregnant. If they said “yes” then they were excluded from the study. We have added these details to the Methods section. (Page 5, lines 19-20)

4. *Standard pretested questionnaire to collect sociodemographic data and quantify knowledge and practices of menstruation. – Is the MHM questionnaire used in this study, the standardized and pre validated questionnaire? Or whether the Questionnaire was pretested and validated by the research team of this paper?*

**Authors**: Thank you for your comment. All the tools that we used, were validated previously in other studies, however we adapted them to our local context (when needed) and we also piloted them before collecting the final study data. We have added more information in the Methods section, explaining how we develop the tools, how we tested and how we collected the study data. (Page 7, lines 7-25; Page 8, lines 1-5)

*5. In addition, a modified Beck Depression Inventory II (BDI II) was used to screen for symptoms of depression among the adolescents. BDI II has 21 items with a score range of 0-3 and is a validated screening tool for depression in both adults and adolescents worldwide [27, 28]. A score of > 16 is suggestive of clinical depression [29]. We dropped one item that explore “interest in sex” from our study as this was not culturally appropriate for this setting. Our modified version therefore had 20 items. – What was the validation process adopted after deleting one question from a validated inventory scale? How was the scoring done for depression after deleting one question?*

**Authors**: Thank you. We have added more information in the Method section about the process of adapting the Beck Depression Inventory II (BDI II) to this rural Gambian context. Discussing “interest in sex” among adolescents in this community was a taboo in this community but as this tool has been validated for use in sub-Saharan Africa, we were keen to use it in this exploratory study. (Page 7, lines 11-25) We viewed dropping this one question as a small change that did not require validation, but we appreciate that this approach may limit the generalisability of our findings. We have discussed this under the limitations. (Page 21, lines 9-16)

*6. Mid-stream urine samples were collected for urine dipstick analysis using the Combur-test strips [30] that was performed at the schools. What’s purpose of Combur test strip? – Explain the standard guidelines, protocols and procedures followed for sample collection and the performance of the test in the methodology?*

**Authors**: Thank you. The Combur test strips were used to test the urine for any evidence of a urinary tract infection. We have added the requested information in the methods section and as supplementary material. (Page 6, lines 10-22)

*7. A pre-determined tool was used to assess the WASH hardware available; number and type of toilets, handwashing facilities and disposal facilities. As well as access the quality of these facilities; functionality, gender specificity, privacy, cleanliness, and availability of water and soap. Did the research team informed to the school authorities about these inspection / supervising activities on the facilities beforehand? If yes – How early it was informed to them?*

**Authors**: Thank you for your comment, one unannounced visit to conduct spot check in each school was conducted. Permissions to conduct unannounced visits were obtained from each school at the beginning of the study. We have added this information in the method section. (Page 6, lines 25-26; Page 7, lines 1-4)

Kind regards,

Belen Torondel

Department of Disease Control, Faculty of Infectious Diseases

London School of Hygiene and Tropical Medicine

Keppel St, London WC1E7HT, UK

+44 (0) 20 7636 2934
